# To Be Ethical and Responsible Digital Citizens or Not: A Linguistic Analysis of Cyberbullying on Social Media

**DOI:** 10.3389/fpsyg.2022.861823

**Published:** 2022-04-29

**Authors:** Jinping Zhong, Jing Qiu, Min Sun, Xiunan Jin, Junyi Zhang, Yidong Guo, Xinxin Qiu, Yujie Xu, Jingxiu Huang, Yunxiang Zheng

**Affiliations:** School of Educational Information Technology, South China Normal University, Guangzhou, China

**Keywords:** linguistic analysis, cyberbullying, digital citizen, social media, content analysis

## Abstract

As a worldwide epidemic in the digital age, cyberbullying is a pertinent but understudied concern—especially from the perspective of language. Elucidating the linguistic features of cyberbullying is critical both to preventing it and to cultivating ethical and responsible digital citizens. In this study, a mixed-method approach integrating lexical feature analysis, sentiment polarity analysis, and semantic network analysis was adopted to develop a deeper understanding of cyberbullying language. Five cyberbullying cases on Chinese social media were analyzed to uncover explicit and implicit linguistic features. Results indicated that cyberbullying comments had significantly different linguistic profiles than non-bullying comments and that explicit and implicit bullying were distinct. The content of cases further suggested that cyberbullying language varied in the use of words, types of cyberbullying, and sentiment polarity. These findings offer useful insight for designing automatic cyberbullying detection tools for Chinese social networking platforms. Implications also offer guidance for regulating cyberbullying and fostering ethical and responsible digital citizens.

## Introduction

The development of information and communications technology (ICT), and accompanying popularity of the Internet, mobile phones, and social media platforms, has increasingly led people to socialize online vs. in person. The COVID-19 pandemic has amplified this phenomenon. Figures suggest that more than 4.88 billion people use the Internet worldwide (nearly 62% of the global population), of whom 4.55 billion (57.6%) use social media frequently (DataReportal, 2021). People in China rely heavily on social media sites, such as Weibo (microblogs), WeChat, QQ, Toutiao (Today’s Headlines), and TikTok. A report from China Internet Network Information Center indicated that over 1 billion people in the country use the Internet, accounting for more than 1 in 5 of the world’s Internet user base. Current trends indicate that social media users in China will surpass the equivalent of 60% of the global population in the first half of 2022 (DataReportal, 2021).

However, people’s excessive screen time, insufficient digital knowledge, and poor awareness of rights and responsibilities in cyberspace have spurred cyberbullying on nearly all social media platforms. Cyberbullying refers to aggressive behavior, which may include jokes, threats, and disinformation, that repeatedly harms people (Smith et al., 2008; Patchin and Hinduja, 2010). Social media enables these actions within a convenient environment that attracts a wide audience (Huang and Chou, 2010). Cyberbullying has thus come to pose a new threat to social media users, especially teenagers aged 6–18. Repeated harm from cyberbullying marked by power imbalances can lead victims to display low self-esteem, anxiety, depression, and even suicidal ideation (Olweus, 2012). A growing number of reports (John et al., 2018; BJNEWS, 2021; Limbana et al., 2021) have indicated that cyberbullying brings grave physical and psychological harm to victims. As a serious global problem, cyberbullying has come to the attention of researchers, administrators, teachers, and parents.

To address this cyber threat, many studies—from theoretical analysis to law promulgation—have focused on the topic and ways to detect it. Mining textual information is a common approach and has shown utility in identifying and predicting human behavior (Davahli et al., 2020). Text features extracted from social media posts were found to be significantly correlated with individuals’ characteristics (Farnadi et al., 2016). Numerous studies have attempted to link textual information with human behavior, including in emotional, social, and cognitive respects (Liu, 2012; Gutierrez et al., 2021). For example, Tausczik and Pennebaker (2010) argued that determining the physiological meaning of textual information can provide insight into people’s thought processes, emotional states, intentions, and motivations. Semenov et al. (2010) analyzed users’ social media posts to try to identify potential school shooters. Negative words in users’ comments on social media may also be related to socially aggressive behavior (Gutierrez et al., 2021). The Sapir–Whorf hypothesis suggests that language use influences human behavior, such that a shift in language use can unconsciously influence one’s thoughts and actions (Kihlstrom and Park, 2018).

Many factors influence cyberbullying. Individual-level factors have direct impacts, especially in terms of literacy related to digital citizenship (Zhong et al., 2021). Digital citizenship refers to using technology in a safe, responsible, and ethical manner; the concept is closely related to socializing online. A person’s level of digital citizenship partly determines their awareness, preferences (e.g., word choice), and behavior. Ideally, if all Internet users are qualified digital citizens, then the incidence of cyberbullying should decline substantially. In other words, cyberbullying can be curbed if people are educated to behave at their best; such habits include pondering how technology might affect others (Ribble, 2015). For instance, one should show respect to others online, be cautious when sharing information or opinions, and pay attention to the wording of posts. Given that many people rely heavily on social networking, which is mainly text-based, digital citizenship is mediated through language. Persistent posting behavior (and the accompanying text, as a form of digital footprints) can inform norms and guidance to improve digital citizenship based on fine-grained analysis of social language. This information can help to mitigate unethical behavior, such as cyberbullying.

To this end, we examine people’s use of social language online *via* a linguistic analysis of cyberbullying. Most relevant research has addressed explicit cyberbullying in English contexts (Ying et al., 2012; Ptaszynski et al., 2016; Balakrishnan et al., 2019; Mladenović et al., 2021). Little is known about cyberbullying conducted in Chinese (Li, 2019, 2020; Xu, 2021) or with implicit language (e.g., with positive wording but negative connotations). Ambiguity also pervades Chinese contexts due to polysemy, incompleteness, and abbreviations in sentences. The language is therefore highly likely to be misunderstood or used with ulterior motives, leading to uncertainty or conflict that can gradually evolve into cyberbullying. Therefore, we extract the linguistic features of cyberbullying in a Chinese context from explicit and implicit perspectives on social media to provide guidance for detecting and governing cyberbullying as well as shaping ethical and responsible digital citizens. Specifically, researchers can refer to the study results to formulate automatic cyberbullying detection; administrators can better understand how people behave on social media and develop pertinent guidelines. The findings are also expected to raise the awareness of users, most of whom are digital natives, about ethical standards and codes of conduct on social networks. The following research questions (RQs) guide this work:

RQ1: What are the linguistic features of cyberbullying on social media in the Chinese context?

RQ2: Do cyberbullying incidents occurring in different domains possess distinct linguistic features?

RQ3: What implications do these features have for (a) the detection and governance of cyberbullying and (b) the shaping of ethical and responsible digital citizens?

## Related Work

### Cyberbullying

Cyberbullying is an emerging form of bullying carried out *via* the internet and digital technologies (Diamanduros et al., 2008); it represents an increasingly serious online moral failure in the internet age. Scholars have often defined cyberbullying in relation to traditional bullying (Smith et al., 2008; Patchin and Hinduja, 2010). Olweus (1995) stated that cyberbullying involves repetition, intentionality, and power imbalance. Yet these attributes are subject to change given the nature of the digital world. For example, repeated aggression may not apply to cyberbullying; rather, retweets of and “likes” on an image or video may perpetuate a victim’s bullying experience (Alsawalqa, 2021) and increase exposure through tags and hashtags (Chan et al., 2021). Accordingly cyberbullying can be defined as aggressive behavior (e.g., jokes, threats, and disinformation) intended to harm other people and communities on the internet.

Cyberbullying can take numerous forms, including flaming, harassment, denigration, impersonation, outing and trickery, exclusion, and cyberstalking (Willard, 2007). The most common types are insults, ridicule, provocation, and ostracism. Literal attacks on others are especially frequent on social media. Typical linguistic features of cyberbullying consist of name-calling, denigration, and mockery. Such language can lead to adverse social, physical, and psychological effects (Nixon, 2014; John et al., 2018; Martínez-Monteagudo et al., 2020). Even so, cyberbullies rarely realize that their harsh or aggressive behavior could be considered bullying, instead perceiving it as humor (Alsawalqa, 2021).

Many methods have been proposed to detect cyberbullying. Machine learning and natural language processing (NLP) techniques are typically used for automatic detection by matching textual data with identified features. Researchers initially applied the bag-of-words approach, part-of-speech tagging, n-gram features, or a combination thereof for feature detection (Dinakar et al., 2011). Most recent studies have focused on content-based features, such as lexical, syntactic, and sentiment information; findings have demonstrated the importance of these words in the automatic detection of cyberbullying (Ptaszynski et al., 2016; Zhao et al., 2016; Zhao and Mao, 2017; Perera and Fernando, 2021).

Even with these advances, cyberbullying detection is inherently difficult and extends beyond simply discerning the negative sentiments or abusive content in a message (Ptaszynski et al., 2016). Online forms of communication are prone to misinterpretation (Tan, 2019), and not all bullying consists of insults (Li, 2020). Additionally, words can be masked (e.g., through metaphors, homophones, and abbreviations) to obscure negative expressions or profanity (Chen et al., 2013; Caselli et al., 2020). Tan et al. (2019) highlighted that spelling alterations are prevalent in cyberbullying, as people tend to simplify words to avoid being caught by the system. Cyberbullying can thus be classified as either explicit or implicit depending on clarity (Tan, 2019; Zeng et al., 2019; Caselli et al., 2020; Li, 2020). In outlining which words did and did not indicate bullying, Waseem et al. (2017) distinguished abusive language by its degree of explicitness. Li (2020) classified words into a cyberbullying word category and sensitive cyberbullying category. Explicit cyberbullying language has a clear negative meaning and no hidden meaning; implicit cyberbullying language often contains ambiguous words, sarcasm, and/or an absence of profanity or hateful terms (Waseem et al., 2017). Existing methods can only identify specific types of cyberbullying, such as threats, sexual harassment, and aggression (Chatzakou et al., 2017; Hee et al., 2018); sarcasm and euphemisms are more difficult to detect (Dinakar et al., 2011). The rapid evolution of Internet language will affect keyword-based cyberbullying detection as well (Ali et al., 2018; Tan, 2019).

Given the limitations of relevant studies, meta-information—covering characteristics, such as a user’s age, gender, location, and posting history—has been considered for cyberbullying detection (Al-garadi et al., 2016; Chatzakou et al., 2017; Hee et al., 2018). More remains to be learned about the linguistic attributes of cyberbullying in addition to expanding the dimensions of and approaches to detection. Much of the extant cyberbullying detection literature has addressed linguistic features; however, a lack of clarity persists around linguistic characteristics and their meanings in this context.

### Linguistic Features of Cyberbullying

Cyberbullying represents a language-related problem in interpersonal communication. The language used online reflects people’s internal thoughts, emotional states, and intentions (Habsah et al., 2016) and may contain directly or indirectly offensive words (Fortuna and Nunes, 2018). Cyberbullying is conventionally detected based on linguistic features. Early researchers used n-grams, the bag-of-words approach, and similar techniques to make coarse-grained predictions about cyberbullying content (Dinakar et al., 2011; Reynolds et al., 2011) by analyzing certain linguistic features (e.g., personal words, pronouns). Grammatical and sentimental features have been widely used more recently (Zhao and Mao, 2017; Hee et al., 2018), suggesting the utility of lexical features for cyberbullying detection.

Most studies on cyberbullying detection revolve around two linguistic attributes: lexical features and grammatical features. In terms of lexicality, a trademark of cyberbullying is a high density of vulgar words (Ptaszynski et al., 2016). Most offensive sentences include not only offensive words but also user identifiers (i.e., second-person pronouns, the victim’s screen name, and other person-centered terms). Punctuation, such as exclamation points, can also predict offensive content by indicating users’ feelings or volume of speaking (Ying et al., 2012). Nobata et al. (2016) summarized 13 types of linguistic features to identify abusive language, such as the number of polite expressions and modal words in text. The politeness principle posits that one’s politeness can be measured by the extent of indirectness in discourse; that is, the number of indirect words can be used to evaluate the degree of euphemism and credibility in a sentence. Regarding grammatical features, syntactic characteristics (e.g., dependency relationships between words) are of primary interest. The linguistics of cyberbullying involve the tone and syntax of speech. Scholars have found that speakers who frequently use imperative sentences tend to be more insulting as they deliver stronger sentiments (Ying et al., 2012). Text length can also predict cyberbullying (Nobata et al., 2016). Ying et al. (2012) argued that user-level features (e.g., one’s writing style, posting patterns, or reputation) can improve the cyberbullying detection rate.

Linguistic forms of cyberbullying can be influenced by cultural contexts (Saengprang and Gadavanij, 2021). Much of the associated literature has analyzed linguistic features of cyberbullying in Western cultures, especially in English; few studies have concentrated on non-English language cyberbullying in Eastern cultures. Saengprang and Gadavanij (2021) compared the linguistic features of cyberbullying between the United Kingdom and Korea. They discovered that indirect speech acts, usually manifesting as one’s adoption of the interrogative mood, were more common in Eastern settings than direct speech acts. Zhang et al. (2019) found that bullying words were useful for classifying cyberbullying in Japan, with informal language and emerging words in tweets affecting the results of sentiment analysis. Research from Pakistan showed that cyberbullies attacked the victim’s appearance through comparisons and certain discourse markers (e.g., capitalization, punctuation, and mathematical symbols; Rafi, 2019). Tan et al. (2019) examined linguistic features of cyberbullying among Malaysian youth from the perspectives of victims, perpetrators, and bystanders. Results indicated that the words these groups used spanned three categories of insults: intellect, physical appearance, and value. Also in Malaysia, language use was found to correlate with people’s intentions: insults did more than degrade and belittle in self-deprecating body-shaming posts; insults also helped posters save face and reduced backlash from other netizens (Tan, 2019). Mohd et al. (2021) revealed that the profane words used in different cyberbullying roles were somewhat similar but featured distinct weights and percentages, which could guide cyberbullying detection. In the Chinese language specifically, a linguistic analysis of a Chinese cyberbullying incident revealed that bullies tended to use negative words, derogatory nouns, and more second-person pronouns (e.g., “you”) or the victim’s real name to accuse the victim. In terms of sentence patterns, posters tended to use exclamatory sentences to convey a certain emotion and use affirmative sentences to judge the victim (Xu, 2021). Li (2019) divided insulting words on Weibo into levels of offensiveness; for example, words in Level 5 were inherently insulting and widely used, whereas those in Level 1 were context-dependent. However, not all cyberbullying comments contain directly offensive words. Terms can be further classified as cyberbullying words (e.g., abusive words, sexual words, and swear words) or as sensitive cyberbullying (e.g., emotional words, emphatic and cathartic words, newly emerging words, idiomatic set phrases, and ordinary words with special meanings; Li, 2020). Li (2020) additionally discovered that cyberbullying words in Chinese and English differed in the use of verbs, adjectives, and nouns. Overall, Chinese cyberbullying words appear more complex than those in English.

### Cyberbullying and Digital Citizenship

Cyberbullying is a form of online anomie related to technology misuse, spurred by the ubiquity of the Internet and social networking. Cyberbullying incidents are tied to a lack of digital citizenship education: many people are unaware of how to use technology safely, legally, and responsibly and lack an adequate understanding of what constitutes sound digital citizenship. Unsurprisingly, individuals can be less inhibited and present a unique virtual self under ineffective supervision without realizing whether their behavior has hurt others. A growing number of people are misusing technology or using it freely to the neglect of others’ feelings. Confrontation and even cyberbullying have thus become unavoidable. In essence, cyberbullying on social media is closely related to one’s level of digital citizenship (Zhong et al., 2021).

From a digital citizenship standpoint, refraining from cyberbullying is an important social skill. The International Society for Technology in Education (ISTE) defines a digital citizen as a person who “recognizes the rights, responsibilities, and opportunities of living, learning and working in an interconnected digital world and acts and models in ways that are safe, legal and ethical” (Brooks-Young, 2017; International Society for Technology in Education, 2019). It is crucial to respect others and to protect oneself and others (Ribble, 2015) when using online social networks. Digital citizenship education is crucial to this aim and has become popular in many countries (e.g., the United States, Singapore, and Australia). We found cyberbullying to be a required module in many online courses, including *Cyberbullying, Digital Drama & Hate Speech* from Common Sense Media; *Ethics and Empathy* from MediaSmarts; and the *Interland* gaming module from Google. Cyberbullying, as a global issue and common online behavior, will likely continue to be a vital aspect of digital citizenship education.

Cyberbullying entails three elements of digital citizenship: digital etiquette, digital law, and digital rights and responsibilities (Ribble, 2015). Instead of merely improving existing laws and regulations, cultivating ethical digital natives can more effectively combat cyberbullying. Researchers have conducted empirical investigation (Chai et al., 2013; Abd Rahman et al., 2014) but have paid limited attention to devising specific behavioral guidelines (Anderson, 2016; Mangkhang and Kaewpanya, 2021). In a digital society, the civility of language is the most direct and explicit manifestation of a person’s level of digital citizenship. Digital citizenship education, supplemented with online social standards based on linguistic analysis, will likely be conducive to developing qualified digital citizens.

## Methodology

**Figure 1:**
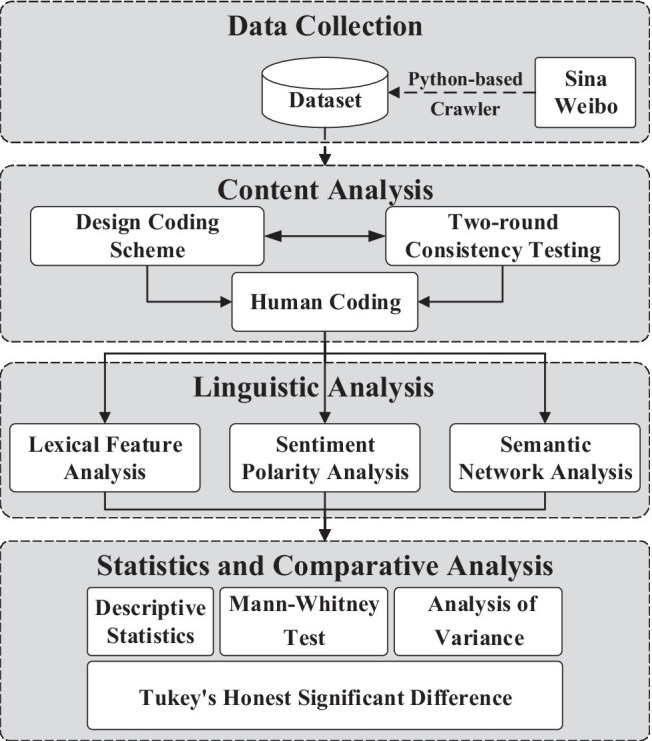
Methodological framework.

As depicted in Figure 1, we applied a four-step methodology to explore the linguistic characteristics of cyberbullying on Chinese social media. We first gathered data from Sina Weibo using a Python-based web crawler. We next established a MySQL database to store the dataset after removing blank text and emojis. Each data record contained the following information: user ID, comment content, posting time, and other post-related information. Second, 23,980 comments were extracted from the database *via* stratified sampling, after which we performed content analysis to sort selected comments and discern users’ intentions in cyberbullying incidents. Third, to address RQ1 and RQ2, the categorized comments were fed as input into a data preprocessing program and submitted for linguistic analysis using traditional natural language processing techniques. Linguistic analysis consisted of three steps: lexical feature analysis, sentiment polarity analysis, and semantic network analysis. The analysis results were then compared in SPSS.

### Data Collection

To avoid interference from invalid or unclear data, the following criteria were applied to choose representative cyberbullying incidents: (1) generality, such that the incidents related to several aspects of daily life; (2) time validity, ensuring that the chosen incidents were timely (i.e., had occurred no earlier than 2019); and (3) notability, in that the incidents aroused widespread public concern (i.e., as indicated by more than 500 retweets). We searched Sina Weibo using these criteria and identified five cyberbullying incidents that spanned multiple domains of social life, including education, entertainment, society, finance, and sports. All incidents took place between October 2020 and October 2021 and collectively garnered more than 100 million reads and over 60,000 discussions. Posts and comments about each incident were jointly screened and grabbed by a Python-based crawler we developed. These data were stored in MySQL database tables, labeled with the original hashtag referring to each incident. Our dataset included 43,111 elements; detailed information is listed in Table 1.

**Table 1:** Basic information about selected cyberbullying incidents.

Identifier	Domain	Public concern	Summary	Conflict focus	Random sampling
Case 1	Education	1.44 Billion reads; 272,000 discussions	On November 20, 2020, a female student from the Academy of Arts and Design of Tsinghua University claimed that a male student had harassed her and then publicized his private information on social media, causing the male student to be cyberbullied. A subsequent check of the video recording revealed a misunderstanding: the man had not touched the woman at all. Although the woman clarified the situation immediately, the incident continued to be reposted and gained wide attention on Sina Weibo.	Gender antagonism	8,185
Case 2	Entertainment	980 Million reads; 128,000 discussions	On January 14, 2021, a singer from Tianhao Shengshi Entertainment Company called Y (youngest daughter of the president of a famous company) announced her formal debut under the label “Unconventional Princess.” Her father’s company was facing pressure from international politics at the time. The video of her interview drew extensive criticism from viewers, as Y had publicly expressed jealousy toward her older sister. She began to be bullied and was forced to stay indoors.	Gap between rich and poor	10,337
Case 3	Society	370 Million reads; 67,000 discussions	On August 5, 2020, Mr. T was diagnosed with COVID-19. His profile and that of a close contact were spread online, along with their epidemiological survey records, minutes later. They were accused of endangering public safety, with some people even claiming that they were a couple and had their own sexual partners. Mr. T was dubbed “Wuhan Hai Wang” and was ridiculed by many netizens. He later stated that the rumors were not true.	Personal privacy and public safety	3,669
Case 4	Finance	570 Million reads; 77,000 discussions	In November 2020, an Internet celebrity known as Mr. X sold a company-produced bird’s nest *via* a livestream. Consumers later questioned whether the product was a fake replica. They were extremely angry and abused Mr. X on Sina Weibo. Finally, Mr. X recalled the product and made a payout.	Disputes between consumers and businesses	5,919
Case 5	Sports	250 Million reads; 84,000 discussions	At the 2021 Tokyo Olympics, Japanese table tennis player Miss M and her partner defeated the Chinese team in a match. However, Chinese audiences heavily ridiculed and spoofed her unusual facial expressions and posture on social media.	National and religious contradictions	15,001
Total					43,111

### Content Analysis

Content analysis is an umbrella term for forms of textual analysis that typically involve ranking, comparing, and categorizing a diverse collection of data (Schwandt, 1997). In previous studies, content analysis was generally used to distinguish cyberbullying content (Saengprang and Gadavanij, 2021), in order to provide training data for machine learning (Dinakar et al., 2011) or to identify cyberbullying vocabulary (Li, 2019, 2020). We adopted content analysis to provide a holistic view of cyberbullying on social media. This analytical approach is based on a well-designed coding scheme; as such, we devised a two-dimensional scheme to categorize our dataset and to capture the overall characteristics of cyberbullying. Coding dimensions included verbal meaning (i.e., explicit bullying, implicit bullying, and non-bullying) and speech intention (i.e., supporting, opposing, and neutral). Explicit bullying is associated with negative connotations and aggression, such as insults, attacks, curses, threats, and sexual harassment (Chatzakou et al., 2017; Hee et al., 2018). Implicit bullying often takes complex linguistic forms that disguise cyberbullying behind instructional, persuasive, speculative, judgmental, imputed, and exaggerated language (Zeng et al., 2019). Non-bullying refers to comments that are unrelated to the incident, used to comfort the victim, or are rational. To promote a more in-depth analysis, we also considered speech intention as proposed in speech act theory (Austin, 1962), which posits that speakers express their intentions *via* utterances (Saengprang and Gadavanij, 2021). The three types of verbal meaning and speech intentions reflected nine comment types in our dataset and constituted the preliminary coding scheme.

Next, seven trained coders were invited to perform content analysis on our dataset using the above scheme. Analysis proceeded through two phases. In the reliability testing phase, we conducted a two-round consistency test to prevent errors caused by an inconsistent understanding of cyberbullying (see Figure 1). Coders were grouped by case in each round and coded the content of sample data (2% of the full sample) separately, after which inter-rater reliability was examined by calculating Cohen’s Kappa coefficient. We next revised our coding scheme based on this coefficient and coders’ feedback to enhance quality. In the formal coding phase, a random sample of selected posts and comments was manually coded (*n* = 23,980, accounting for 55.6% of the full sample), with inter-rater agreement computed as shown in Table 2. The average Kappa coefficient was greater than 0.86, indicating significantly high consistency among coders.

**Table 2:** Consistency test results.

Case	Composite reliability	
	Round 1	Round 2
Case 1	0.77	0.87
Case 2	0.76	0.90
Case 3	0.75	0.92
Case 4	0.90	0.86
Case 5	0.78	0.92

### Linguistic Analysis

Figure 1 depicts the three steps of linguistic analysis: lexical feature analysis (Step 1), sentiment polarity analysis (Step 2), and semantic network analysis (Step 3). The aim of Step 1 is to explore the lexical features of cyberbullying on Chinese social media. Previous studies used Linguistic Inquiry and Word Count (LIWC) to identify these features of cyberbullying. For example, Hosseinmardi et al. (2015) explored the pattern of linguistic and psychological measurements of four cyberbullying classes through their LIWC values; Singh et al. (2017) found that text-based features outperformed visual features; Salminen et al. (2020) investigated hateful and non-hateful language by LIWC. In this study, we employed a computational linguistic tool called TextMind (Gao et al., 2013), which was developed based on the 2007 version of the LIWC application and C-LIWC (Chinese LIWC) and offers an all-in-one solution from automatic Chinese word segmentation to psychological analysis. One benefit of TextMind is that it provides greater coverage of popular Chinese words that are trending on social media, thus enabling effective textual analysis in Chinese. This tool is also compatible with LIWC2007 and C-LIWC. It defines five general categories of linguistic variables (i.e., linguistic processes, psychological processes, personal concern, spoken categories, and punctuation categories) containing 101 linguistic variables in total. These variables reflect different levels of language use from simple (e.g., word count, use of dictionary words, and number of hashtags) to complex (e.g., psychological constructs and tone of voice). TextMind automatically calculated the proportion of total words in each comment that matched each dictionary category. Results were stored in a CSV file, which allowed for further statistical analysis and data visualization in R or SPSS.

Scholars have deemed sentiment a distinguishing trait among bullies, victims, and non-bullies (Dani et al., 2017; Rosa et al., 2019; Balakrishnan et al., 2020). Most of their sentiment analysis was implemented using NLP (Maskat et al., 2020; Almutairi and Al-Hagery, 2021). Drawing upon such work, we performed sentiment analysis in Step 2 to quantify positive and negative comments. Each comment’s sentiment polarity was analyzed *via* an open and corpus-based application program interface (API) for NLP provided by Baidu, a Chinese artificial intelligence service platform. This API can report affective scores and determine sentiment polarity categories (i.e., positive, negative, and neutral) of various types of content. We accessed the API in accordance with APPID, API Key, and Secret Key and input each comment as the URL request data for sentiment analysis. Results revealed comments’ sentiment polarity categories and probability distribution (e.g., positive prob).

The effectiveness of NLP based on semantic models in cyberbullying detection has been confirmed previously, such as latent semantic index (LSI) and late semantic analysis (LSA; Zhao et al., 2016; Zhao and Mao, 2017). Semantic network analysis can be leveraged to explore group awareness in cyberbullying incidents (Xiong et al., 2019), which is highly applicable to our study given the aim to quantify cyberbullying and non-bullying comments related to specific incidents. In Step 3, we used the software program ROST Content Mining (ROSTCM) to extract high-frequency keywords and generate co-occurrence networks. Different from most semantic network analysis programs which can only analyze English words, ROSTCM is specifically intended for Chinese semantic network analysis and has been widely used in the social sciences (Shen, 2008).

### Statistical and Comparative Analyses

Following content analysis and linguistic analysis, data were fed as input into SPSS for statistical tests. To address RQ1 and RQ2, we first tested the distribution of variables for normality using the Kolmogorov–Smirnov test, which revealed a non-normal distribution (*p* < 0.01). The Mann–Whitney U test was subsequently conducted to determine which linguistic features strongly differentiated cyberbullying; comments’ cyberbullying categories (i.e., explicit bullying vs. implicit bullying) were entered as dependent variables, and linguistic features were entered as independent variables. We next performed analysis of variance on the sentiment polarity variables followed by Tukey’s honest significant difference test to compare cyberbullying incidents’ sentiment polarity. Ten pairwise comparisons were carried out across the five cyberbullying incidents. The statistical results are described in Results section.

## Results

### Content Analysis

Table 3 summarizes the descriptive statistics of content analysis. Approximately 55% of comments in all cases were classified as cyberbullying. People mainly attacked the victims through implicit bullying (accounting for more than 46% of the data); explicit bullying was the least common, appearing in 10.75% of the data. Some seemingly neutral posts also involved bullying, totaling roughly 15% on average in each case.

**Table 3:** Descriptive statistics of content analysis.

Case	Domain	Speech intention	Categories of language			*n*
			Explicit	Implicit	Non-bullying	
Case 1	Education	Supporting	22	89	110	4,782
		Opposing	676	1,675	292	
		Neutral	154	455	1,309	
		Total	852	2,199	1,711	
			17.82%	46.40%	35.78%	
Case2	Entertainment	Supporting	8	10	810	5,075
		Opposing	357	1,962	225	
		Neutral	18	134	1,551	
		Total	383	2,106	2,586	
			7.55%	41.50%	50.96%	
Case 3	Society	Supporting	22	53	371	3,552
		Opposing	120	616	79	
		Neutral	38	702	1,551	
		Total	180	1,371	2,001	
			5.07%	38.60%	56.33%	
Case 4	Finance	Supporting	42	192	679	5,346
		Opposing	378	1,443	180	
		Neutral	117	583	1,732	
		Total	537	2,218	2,591	
			10.04%	41.49%	48.47%	
Case 5	Sports	Supporting	0	0	86	5,225
		Opposing	609	1,691	130	
		Neutral	18	1,296	1,395	
		Total	627	2,987	1,611	
			12%	57.17%	30.83%	
Grand total			2,579	10,901	10,500	23,980
			10.75%	45.46%	43.79%	

Across the five cases, Case 5—with national antagonism as the conflict focus—had the highest rate of cyberbullying, with nearly 70% of posts related to violence. Case 3 concerned personal privacy and public safety; this incident elicited the most neutral views, as over half (56.33%) of comments did not mention violence. The gender antagonism case (Case 1) was most clearly associated with cyberbullying: nearly 18% of comments included explicit bullying.

### Lexical Feature Analysis

#### Comparison of Cyberbullying and Non-bullying Comments

Results of the linguistic analysis for all cases are summarized in Table 4. Marked differences were observed among comment types in all linguistic feature dimensions. Regarding linguistic processes, cyberbullying comments had significantly higher word counts, contained more tags, and applied dictionary terms involving personal pronouns (e.g., second-person pronouns and third-person singular pronouns), swear words, and adjunct words (e.g., auxiliary verbs, prepositions, quantifiers, tense markers, and numbers). Non-bullying comments comparatively contained more functions and multi-functional words, interjections, personal pronouns (e.g., first-person plural pronouns) and impersonal pronouns, and other words used as modifiers (e.g., negations, verbs, adverbs, and conjunctions).

**Table 4:** Linguistic features of language categories with significant differences in all cases.

Categories	Cyberbullying		Non-bullying		*p*	Explicit		Implicit		*p*
	M	SD	M	SD		M	SD	M	SD	
**Linguistic processes**										
WordCount	13.85	15.689	13.228	16.617	**	13.157	14.935	16.778	18.255	**
Word PerSentence	9.061	8.767	8.641	9.239	**	8.844	8.574	9.980	9.486	**
Rate DicCover	0.789	0.208	0.768	0.263		0.791	0.213	0.781	0.188	**
Rate numeral	0.006	0.048	0.008	0.070	**	0.006	0.050	0.006	0.038	**
Words > 6 letters	0.003	0.040	0.008	0.069	**	0.004	0.044	0.001	0.021	
Words > 6 letters	0.027	0.111	0.051	0.182		0.030	0.118	0.015	0.071	**
Rate LatinWord	0.013	0.066	0.02	0.103		0.013	0.070	0.011	0.046	**
Num HashTag	0.007	0.123	0.004	0.089		0.006	0.127	0.009	0.100	*
Funct	0.375	0.212	0.381	0.245	*	0.370	0.216	0.393	0.195	**
Pronoun	0.074	0.094	0.068	0.104	**	0.072	0.094	0.085	0.092	**
PPron	0.045	0.078	0.038	0.073	**	0.044	0.079	0.048	0.073	**
We	0.001	0.011	0.002	0.015	**	0.001	0.011	0.001	0.007	
YouS	0.013	0.042	0.01	0.039	**	0.012	0.042	0.016	0.044	**
SheHe	0.012	0.04	0.008	0.033	**	0.011	0.038	0.018	0.05	**
iPron	0.030	0.06	0.031	0.077	**	0.029	0.059	0.038	0.063	**
Verb	0.110	0.119	0.125	0.143	**	0.108	0.12	0.122	0.116	**
AuxVerb	0.025	0.058	0.023	0.058	**	0.025	0.058	0.026	0.055	**
Adverb	0.089	0.108	0.104	0.133	**	0.088	0.109	0.091	0.101	**
Preps	0.032	0.065	0.031	0.067	*	0.032	0.066	0.033	0.057	**
Conj	0.028	0.056	0.030	0.057		0.028	0.057	0.029	0.053	**
Negate	0.002	0.015	0.002	0.020		0.002	0.016	0.002	0.014	*
Quant	0.015	0.046	0.013	0.045	**	0.014	0.045	0.019	0.048	**
Number	0.008	0.031	0.007	0.035	**	0.008	0.031	0.008	0.032	*
Swear	0.005	0.043	0.002	0.027	**	0.003	0.033	0.012	0.070	**
YouPL	0.001	0.008	0.001	0.008	**	0.001	0.008	0.001	0.009	**
PrepEnd	0.009	0.032	0.011	0.042		0.009	0.033	0.009	0.029	**
SpecArt	0.005	0.026	0.006	0.027		0.005	0.027	0.005	0.022	**
QuanUnit	0.02	0.051	0.019	0.058	**	0.019	0.051	0.024	0.052	**
Interjunction	0.102	0.109	0.108	0.133		0.104	0.111	0.093	0.096	**
MultiFun	0.071	0.092	0.074	0.100		0.068	0.091	0.083	0.099	**
TenseM	0.05	0.086	0.042	0.086	**	0.053	0.089	0.037	0.066	**
PastM	0.002	0.013	0.003	0.022	**	0.002	0.013	0.002	0.010	
PresentM	0.005	0.022	0.005	0.024	*	0.005	0.022	0.005	0.020	**
FutureM	0.005	0.023	0.006	0.032	*	0.004	0.023	0.005	0.026	*
ProgM	0.038	0.079	0.028	0.071	**	0.041	0.083	0.025	0.056	**
tPast	0.001	0.013	0.002	0.016	**	0.001	0.013	0.001	0.011	
tNow	0.002	0.012	0.002	0.014		0.002	0.012	0.002	0.012	*
tFuture	0	0.006	0.001	0.013	**	0	0.005	0.001	0.007	
**Psychological processes**										
Social	0.071	0.100	0.084	0.126	**	0.068	0.100	0.081	0.098	**
Family	0.007	0.032	0.005	0.033	**	0.007	0.033	0.007	0.031	**
Humans	0.018	0.052	0.019	0.051		0.017	0.048	0.025	0.066	**
Affect	0.109	0.182	0.088	0.164	**	0.118	0.192	0.075	0.128	**
PosEmo	0.072	0.165	0.058	0.148	**	0.084	0.179	0.024	0.068	**
NegEmo	0.028	0.087	0.019	0.07	**	0.025	0.084	0.039	0.099	**
Anx	0.003	0.029	0.002	0.023	*	0.004	0.031	0.003	0.019	
Anger	0.008	0.046	0.004	0.033	**	0.007	0.041	0.015	0.064	**
Sad	0.002	0.019	0.001	0.016	**	0.002	0.02	0.002	0.014	
CogMech	0.169	0.146	0.196	0.182	**	0.173	0.15	0.154	0.129	**
Insight	0.016	0.044	0.029	0.077	**	0.017	0.046	0.015	0.039	
Cause	0.010	0.033	0.011	0.040		0.01	0.034	0.01	0.030	**
Discrep	0.023	0.054	0.026	0.064	*	0.023	0.056	0.021	0.045	
Tentat	0.020	0.051	0.025	0.065	**	0.02	0.053	0.018	0.043	*
Certain	0.020	0.067	0.03	0.093	**	0.02	0.068	0.019	0.061	*
Inclusive	0.025	0.052	0.027	0.062		0.024	0.053	0.025	0.049	**
Exclusive	0.029	0.056	0.031	0.064		0.028	0.056	0.031	0.054	**
Percept	0.019	0.056	0.021	0.061		0.019	0.056	0.02	0.057	**
See	0.007	0.036	0.006	0.035	**	0.007	0.035	0.008	0.041	**
Hear	0.005	0.025	0.008	0.033	**	0.005	0.025	0.005	0.026	**
Feel	0.003	0.022	0.004	0.027		0.003	0.022	0.003	0.020	**
Bio	0.026	0.073	0.018	0.064	**	0.022	0.065	0.044	0.098	**
Body	0.014	0.051	0.006	0.033	**	0.012	0.046	0.025	0.068	**
Health	0.005	0.039	0.002	0.020	**	0.004	0.031	0.012	0.062	**
Sexual	0.004	0.028	0.003	0.034	**	0.003	0.025	0.007	0.039	**
Ingest	0.005	0.029	0.007	0.042		0.005	0.029	0.005	0.027	**
Relative	0.066	0.096	0.068	0.112	*	0.062	0.093	0.081	0.107	**
Motion	0.017	0.048	0.018	0.060		0.017	0.049	0.018	0.048	**
Space	0.034	0.069	0.031	0.073	**	0.03	0.063	0.049	0.087	**
Time	0.018	0.049	0.023	0.062	**	0.019	0.05	0.018	0.042	**
Psychology	0.017	0.064	0.024	0.085	**	0.017	0.067	0.017	0.052	**
**Personal concern**										
Work	0.025	0.064	0.033	0.083	**	0.026	0.065	0.023	0.061	
Achieve	0.01	0.038	0.012	0.045	**	0.011	0.04	0.006	0.027	**
Leisure	0.019	0.054	0.020	0.063		0.02	0.056	0.014	0.044	*
Home	0.002	0.019	0.001	0.013		0.002	0.019	0.002	0.019	**
Money	0.01	0.039	0.007	0.037	**	0.011	0.041	0.007	0.028	**
Religion	0.002	0.017	0.004	0.026	**	0.002	0.017	0.002	0.014	**
Death	0.008	0.039	0.002	0.014	**	0.006	0.035	0.013	0.053	**
**Spoken category**										
Assent	0.135	0.22	0.087	0.145	**	0.15	0.237	0.069	0.101	**
Nonfl	0.011	0.043	0.014	0.062	*	0.011	0.044	0.011	0.037	**
Filler	0.010	0.032	0.009	0.034	**	0.01	0.031	0.012	0.034	**
**Punctuation**										
Period	0.007	0.029	0.010	0.051	**	0.007	0.030	0.008	0.028	**
Comma	0.037	0.058	0.038	0.062		0.036	0.057	0.043	0.059	**
QMark	0.025	0.086	0.022	0.089	**	0.024	0.086	0.029	0.087	**
Exclam	0.011	0.056	0.009	0.048	**	0.011	0.057	0.011	0.049	**
Parenth	0.001	0.01	0.001	0.015	**	0.001	0.011	0	0.005	
OtherP	0.003	0.027	0.007	0.053	**	0.003	0.028	0.002	0.025	

*p < 0.05; **p < 0.01.

In the psychological processes dimension, cyberbullying comments featured more family words as well as more affective processes and biological processes. The mean value of positive emotion-related words was higher than that of negative emotion-related words in cyberbullying comments. Conversely, non-bullying comments mainly revolved around psychology, cognitive processes, perceptual processes, and terms indicating relativity. In the personal attention dimension, cyberbullying included more references to money and death, whereas non-bullying comments referred more to work, achievement, and religion. The spoken category and punctuation category included frequent use of assent words and fillers in cyberbullying with more question marks and exclamation marks. Nonfluencies, periods, parentheses, and other punctuation were more prevalent in non-bullying comments.

#### Comparison of Explicit and Implicit Bullying

Table 4 presents a comparison of comments displaying explicit and implicit bullying. In terms of linguistic processes, comments categorized as explicit bullying contained more words and tags, frequent function words, pronouns (e.g., second- and third-person singular pronouns and impersonal pronouns), common verbs, swear words, temporal words (e.g., present and future markers), and modifiers (e.g., auxiliary verbs, negations, conjunctions, numbers, quantifiers, specific articles, and unit words of quantity). Implicit bullying showed higher rates of dictionary words and complex words along with more second-person plural pronouns, interjections, and tense markers (e.g., past and progressive markers).

In the psychological processes dimension, explicit bullying typically involved the following: more words about social processes (e.g., family and humans), affective processes (with mainly negative words), perceptual processes, and biological processes (e.g., regarding one’s health, body, or sexuality); inclusive and exclusive words; and words about motion and space in relativity. Implicit bullying contained more positive emotion-related words and words about feeling, psychology, and cognitive processes (e.g., involving causation, tentativeness, and certainty).

The current concerns dimension included more words related to home, religion, and death in explicit bullying but more words about work, family, and leisure in implicit bullying. In the spoken category and punctuation category, the rates of assent words, nonfluencies, and exclamation points were higher in explicit bullying whereas fillers and punctuation (e.g., periods, commas, and question marks) were more common in implicit bullying.

#### Linguistic Features in Different Cyberbullying Incidents

As mentioned earlier, the proportions of cyberbullying categories varied by incident. In the case of education, the victim was cursed with a plethora of death-related words, such as “社会性死亡” (“social death”) and “死” (“die”). The victim was also body shamed, such as when commentors called her “腚” (“butt”). The feminist community was directly abused using swear words including “母狗” (“bitch”) and “犬” (“dog”). In implicit bullying, comments mainly expressed instructional and judgmental language by questioning the victim’s behavior; some comments included strong punctuation [e.g., “凭什么要相互道歉?” (“Why apologize to each other?!”). Others used imperative sentences and exclamation points to make declarations, as in “自作自受!” (“You did it to yourself!”) and “必须起诉她!” (“You must be sued!”)].

In the entertainment case, explicit bullying mainly featured negative words, words related to appearance and nationality, and punctuation that denied and strongly questioned the victim, such as “好难听??” (“So hard to hear??”) and “什么剬主?中国不欢迎美国剬主” (“What princess? American princesses are not welcome in China”). Most implicit bullying was expressed through sarcasm and judgmental language; for instance, “爸爸有钱真好” (“It’s lucky that your father is rich”) sarcastically described the victim’s family background and implied that she had relied on her family’s help to become a star. Comments, such as “方脸不适合出道” (“Square face is really not suitable to be a star”) and “天赋还是很重要的, 你还是算了吧” (“Talent is still very important, you should just give up”), emphasized celebrity-related stereotypes, thus dismissing the victim.

Similarities emerged between the cyberbullying cases centered on sports and society. In both cases, explicit bullying involved many swear words. People attacked the victim’s looks in the sport case with phrases, such as “表情死贱的” (“What a bitchy expression”) and “太丑了” (“That’s so ugly”). Others reinforced rumors in the society case, as indicated by “肯定是他妈真的啊” (“It must be fucking true!”) and “女的不也睡好几个??” (“Didn’t that girl sleep with several people too?”). As for implicit bullying, emotional terms and exaggeration, such as “哈哈哈” (an onomatopoeia for laughing) and “笑死我了” (“You’re really killing me”), were used in both cases to express happiness while making light of the victim’s pain. Comments like “真是人才” (“What a talent”) conveyed support for microblogs vilifying the victims, indirectly amplifying the negative impact of cyberbullying.

Regarding the finance case, explicit bullying mostly took the form of using animals to refer to the victim and cursing him: “仓鼠” (“hamster”) and “杂种狗” (“mutt”). Individuals engaging in implicit bullying demanded that the government punish the victim severely as indicated by strong phrasing, such as “必须/支持封杀” (“He must be shut out by all media”).

### Sentiment Polarity Analysis

**Figure 2:**
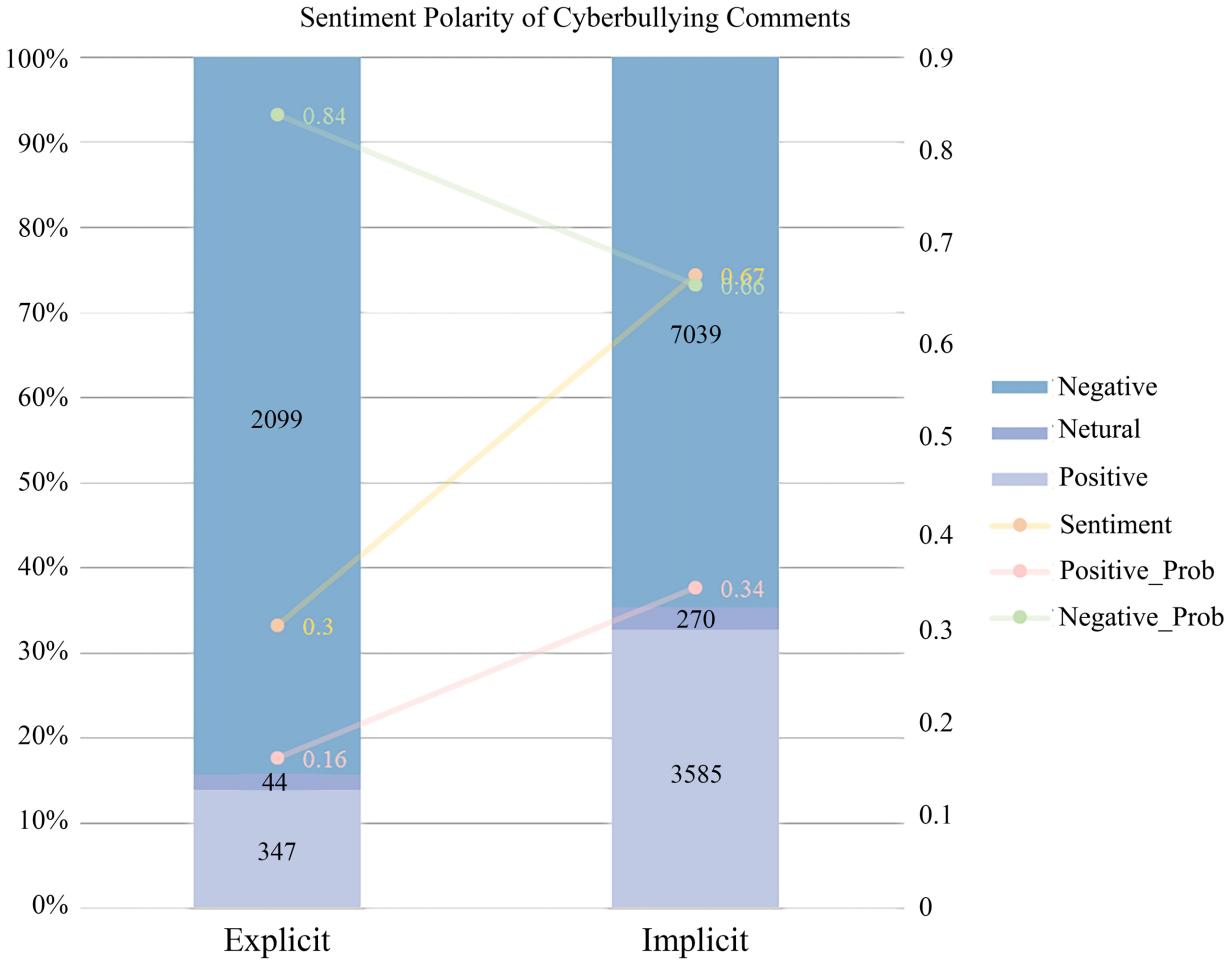
Sentiment polarity of cyberbullying comments (in percentage).

Sentiment analysis was carried out to examine comments’ sentiment polarity as shown in Table 5. The results for different language forms demonstrated significant variation. Generally, cyberbullying was more negative than non-bullying whereas explicit bullying language was more negative than implicit language. Figure 2 illustrates the distribution of sentiment polarity across cyberbullying categories and the overall emotional analysis of samples of implicit bullying and explicit bullying.

**Table 5:** Comparison of sentiment analysis results.

	Cyberbullying		Non-bullying		*p*	Explicit		Implicit		*p*
	M	SD	M	SD		M	SD	SD	M	
Sentiment	0.61	0.907	0.97	0.987	**	0.607	0.907	0.683	0.935	**
Positive_prob	0.6	1.311	0.59	0.862	**	0.603	1.311	0.584	1.207	**
Negative_prob	0.78	0.785	0.65	0.958	**	0.777	0.785	0.758	0.852	**

**p < 0.01.

The sentiment of cyberbullying comments was largely negative: comments classified as explicit bullying (sentiment = 0.30, 84.30% negative) were more negative than those categorized as implicit bullying (sentiment = 0.67, 64.56% negative). Interestingly, nearly 30% of cyberbullying comments were considered positive, presumably due to the adopted implicit bullying tactics.

**Figure 3:**
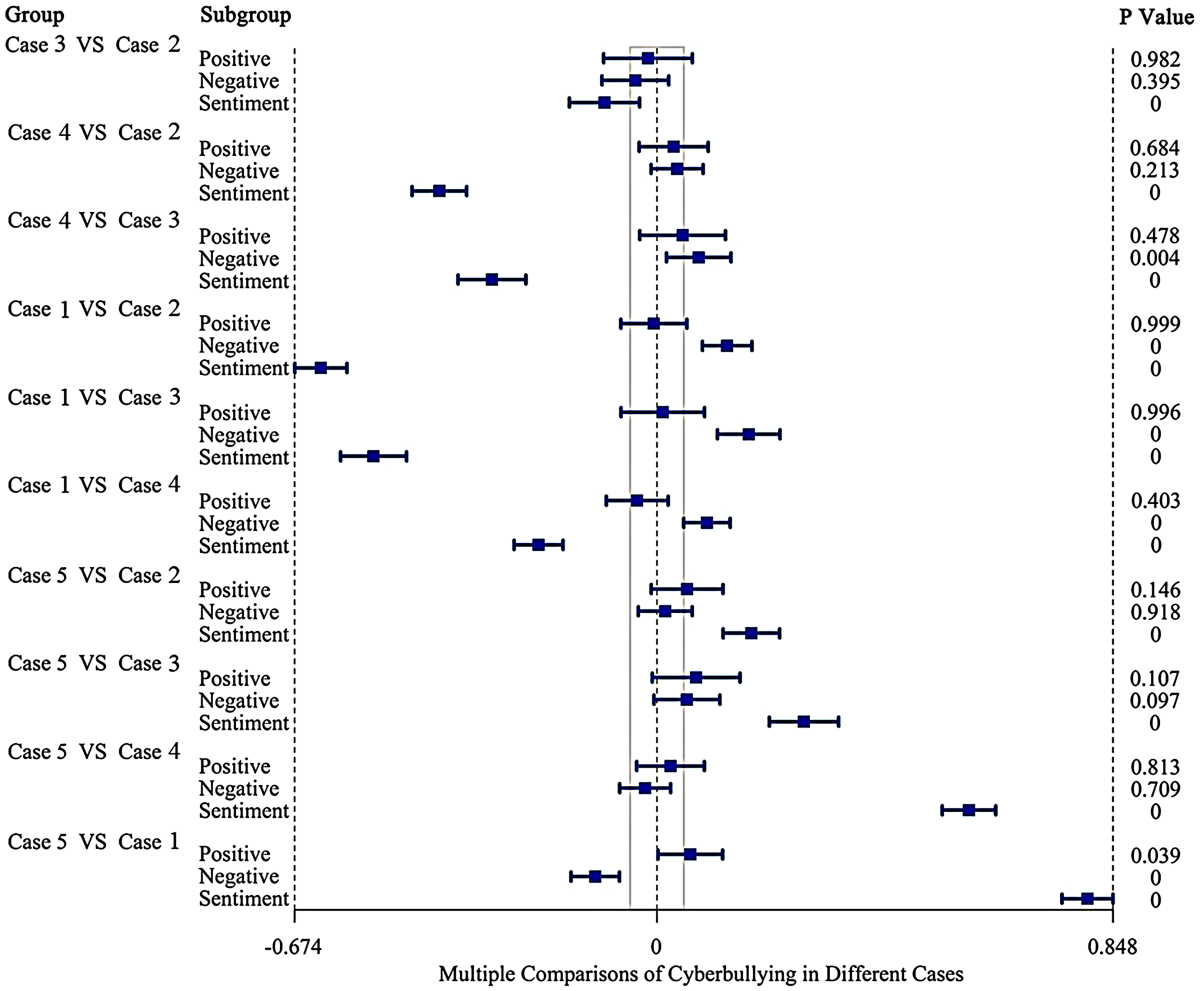
Forest plot of sentiment polarity in cyberbullying cases.

To verify whether sentiment varied significantly between the five cases, we compared cyberbullying comments about different cases (Figure 3). No significant difference manifested in the positive probability, whereas a comparison of Case 1 with other cases in the negative probability revealed significant variation. Essentially, statistical differences were observed in the sentiment of comments for each case compared to others (*p* < 0.001).

Table 6 indicates that Case 1, which was dominated by explicit bullying, had the highest percentage of negative sentiment (84.37%). Nearly half of all comments were positive in Case 5 (46.96%) and Case 3 (42.36%); remarks mainly took the form of implicit bullying, such as ridicule and sarcasm.

**Table 6:** Descriptive statistics of sentiment analysis in different cases.

Cyberbullying comments in different cases		Positive		Neutral		Negative	
		*n*	Percentage (%)	*n*	Percentage (%)	*n*	Percentage (%)
Case 1: Education (*N* = 3,051)	Explicit	99	22.20	10	19.61	743	28.87
	Implicit	347	77.80	41	80.39	1,831	71.13
	Total	446	14.62	51	1.67	2,574	84.37
Case 2: Entertainment (*N* = 2,489)	Explicit	55	9.14	7	8.43	231	13.47
	Implicit	547	90.86	76	91.57	1,484	86.53
	Total	602	24.19	83	3.33	1,715	68.90
Case 3: Society (*N* = 1,551)	Explicit	33	5.02	3	6.38	144	17.00
	Implicit	624	94.98	44	93.62	703	83.00
	Total	657	42.36	47	3.03	847	54.61
Case 4: Finance (*N* = 2,755)	Explicit	55	10.38	10	23.26	473	21.66
	Implicit	475	89.62	33	76.74	1,711	78.34
	Total	530	19.24	43	1.56	2,184	79.27
Case 5: Sport (*N* = 3,614)	Explicit	105	6.19	14	14.14	508	27.94
	Implicit	1,592	93.81	85	85.86	1,310	72.06
	Total	1,697	46.96	99	2.74	1,818	50.30

### Semantic Network Analysis

**Figure 4:**
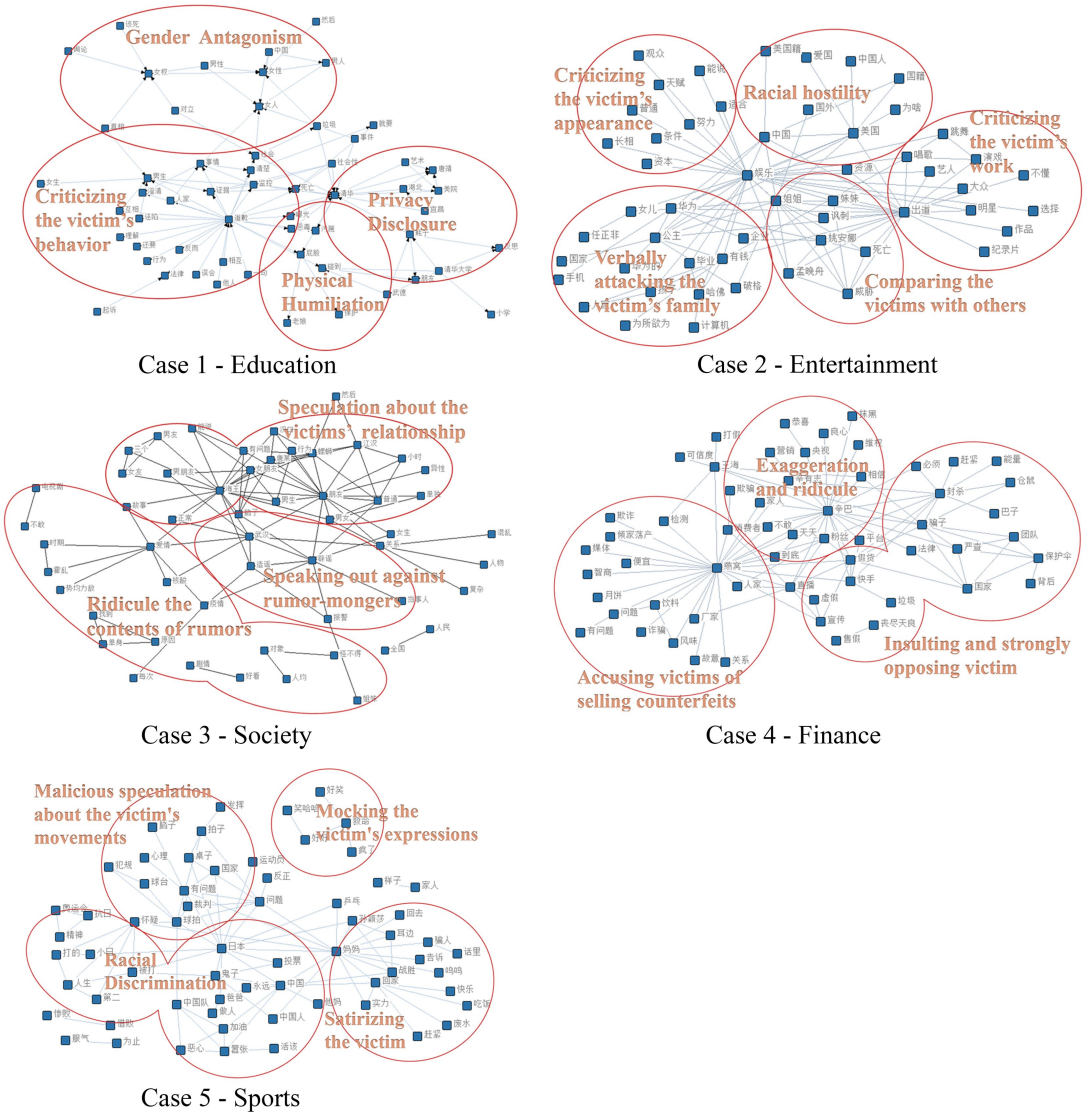
Results of text-semantic network analysis.

Figure 4 displays the results of text-semantic networks. Multiple keywords related to the topic of education involved a series of verbs, gender-based words, and location-related nouns. Perpetrators mainly attacked the victim’s behavior during the incident as well as the victim’s body, education, and profession. In the case focusing on gender antagonism, many commenters insulted feminism and made gender-specific attacks.

Most keywords in the society case covered the following: interpersonal nouns, such as “复杂” (complex), “女友” (“girlfriend”), and “唐某” (“the victim”); words related to rumors, such as “造谣” (“disinformation”) and “报警” (“call the police”); and words involving love and public affairs, such as “疫情” (“epidemic”), “全国” (“nationwide”), and “对象” (“lover”). Bullies in this case speculated maliciously about the victims’ relationship by mocking the content of rumors. Other bullies engaged in verbal abuse to fight against rumor-mongers on the victims’ behalf.

The other three cases shared similar results. Taken together, these findings suggest an association between the use of cyberbullying words and the controversial focus of each incident, with cyberbullies first attacking the victim’s behavior before progressing to attacks on personal privacy, external conditions (e.g., one’s appearance or family), and even a group to which the victim belonged. For example, in the finance case, cyberbullies moved from attacking the victim’s behavior to targeting the livestreaming platform and the victim’s production team. Cyberbullies in the sport case attacked the athlete herself along with her home country.

## Discussion

This study aimed to examine the linguistic features associated with potential cyberbullying (or the likelihood of poor digital etiquette) based on Sina Weibo comments exhibiting instances of explicit bullying, implicit bullying, or non-bullying. Results are addressed based on the three RQs underpinning this work.

### General Linguistic Features of Cyberbullying

According to Zhang and Ghorbani (2020), linguistic characteristics refer to the fundamental components, structure, and semantics of natural language. These aspects cover word-, sentence-, and content-level attributes. This study focused on word- and content-level characteristics to examine their effects on cyberbullying. At the word level, compared to non-bullying comments, cyberbullying often featured a higher word count, a greater number of swear words and adjunct words, more frequent use of second-person pronouns and third-person singular pronouns, more mentions of affective and biological processes, greater references to money and death, and more use of question marks and exclamation points. When compared with explicit bullying, cyberbullying comments involving implicit bullying tended to contain the following: fewer words and tags; greater use of dictionary words and complex words; a higher number of tense markers; fewer swear words and negative emotions; more words related to cognitive processes (e.g., causation and certainty); fewer words related to home, religion, and death; and a greater number of fillers and question marks. These patterns are broadly consistent with those of prior studies (Ying et al., 2012; Nobata et al., 2016; Ptaszynski et al., 2016; Li, 2020; Saengprang and Gadavanij, 2021; Xu, 2021).

Stop words, which are words that carry the least amount of semantic information compared with other words (e.g., “a,” “as”), are usually removed from cyberbullying detection. Cyberbullying comments in our sample contained more function words, prepositions, tense markers, and numbers that could easily be filtered as stop words, implying that stop-word selection can be explored further in the future. This finding is consistent with that of Dewani et al. (2021), who noted that stop words, such as “here” and “today” in some time-sensitive and location-sensitive key messages, could affect NLP results. More subtle cyberbullying may be achieved linguistically through the use of these kinds of words as modifications or similes (Tan, 2019).

Research has shown that personal pronouns can distinguish cyberbullying: personal pronouns and proper nouns are used to identify particular objects (Searle, 1970). Yin et al. (2009) noted that second-person pronouns (e.g., “you” and “yourself”) are more useful than other personal pronouns for harassment detection. Compared with “he” or “she,” “you” evokes a sense of direct interaction—as if the audience were standing in front of the speaker—which limits the force of accusation to the victim (Xu, 2021). Non-bullying comments in this study were accompanied by greater use of first-person plural pronouns, such as “we.” This trend may have arisen because “we” can also function as a politeness strategy in transient interaction and serves to reduce the distance between a speaker and listener (Brown and Levinson, 1987). In cyberbullying incidents, mediators may use “we” to bring others closer to them and to cause readers to be more receptive to mediators’ opinions.

In the social media context, uncertainty leads to inefficient communication and potential cognitive dissonance among the public (Dwivedi et al., 2018). Our results accord with those of Zhou et al. (2021), who identified uncertainty as a salient feature of rumor propagation. Cyberbullies convey uncertainty by using nouns with modifiers (e.g., fillers, expressions of certainty, and tentative and adjunct words). Xu (2021) discovered that derogative nouns (with or without modifiers) enable more negative evaluations of victims, such as by using negations. Cyberbullies also tend to hide their malice behind complex words or nouns with modifiers or by using neutral words to avoid being screened by Internet supervisory authorities.

Our results further showed that, in addition to swear words, emotional words are distinctive features of cyberbullying. Recent studies have indicated that sentiment plays a key role on social media: posters can draw wide public attention by using a large number of emotional words (Guo et al., 2019; Ghanem et al., 2020), which can in turn evoke emotional audience responses (Zhou et al., 2021). Surprisingly, the mean value of positive emotion-related words in our set of cyberbullying comments exceeded that of negative emotion-related words. This outcome was likely due to the prevalence of implicit bullying. In our sample, cyberbullies often used negative words to express their subjective views of an incident; doing so exacerbated responses and amplified associated negative effects. Meanwhile, these posters typically employed positive emotion-related words to satirize and ridicule victims. Group infection theory (Hatfield et al., 1993) maintains that personalized emotions can influence others’ behavior, thoughts, and emotions. This influence can interact and grow among multiple people, driving group members to display uniform emotional states and social perceptions (Bowen and Blackmon, 2003; Smith and Conrey, 2007). Along with using emotive expressions, improper punctuation use is common in emotional expression (Ying et al., 2012; Salminen et al., 2020). Cyberbullies may use certain discourse markers (e.g., punctuation and mathematical symbols) to accentuate their messages (Rafi, 2019). Punctuation abuse was found to be particularly frequent in instances of Chinese cyberbullying, especially overusing exclamation points and question marks. Interrogative sentences are one of the most popular types of indirect speech used to express inquiries or requests (Yule, 1996). Yet the heavy use of question marks in cyberbullying content reflects strong skepticism and is loaded with anger. A creative use of language in cyberbullying appeared in questions containing “为什么” (“why”) that ended with an exclamation point (Xu, 2021). Saengprang and Gadavanij (2021) suggested that the interrogative mood is often used to express the sender’s opinion rather than to ask a question.

### Comparisons of Linguistic Features Across Cyberbullying Incidents

Our findings revealed that cyberbullying varied by incident in terms of three aspects: the linguistic features of cyberbullying, word use, and comment sentiment. Descriptive statistics showed that cyberbullying comments displaying implicit bullying were more prevalent than non-bullying content. As mentioned, netizens tended to express their opposition to the victim through context-sensitive words and indirect language, such as interrogative sentences. Some netizens did not initially intend to attack the victim and may have simply meant to mock seemingly humorous parts of the incident. Doing so could inadvertently feed ridicule and rumors, especially in selected cases involving sport and society. These consequences might have occurred because recreational aggression, which is unprovoked and conducted in order to obtain instant gratification (e.g., a quick thrill), usually motivates cyberbullying. Perpetrators may not focus on the impacts of their behavior and thus may not fully understand these effects (Graf et al., 2022).

To gain a richer understanding of cyberbullying at the content level, we scrutinized the semantic network of words and noticed that the controversial focus of the incident—not the domain—influenced posters’ word choices. Cyberbullying generally begins with controversial behavior, ranging from criticism of the victim’s appearance, experience, and family to indiscriminate attacks on a specific group of victims. Forms of cyberbullying differed significantly across all domains. This result is somewhat challenging to interpret given the lack of a common denominator between areas, but the variation could be attributed to social identities and group categorization. Social identity theory suggests that individuals identify with their own group *via* social categorization, which spurs in-group favoritism and out-group hostility (Tafjel and Turner, 1979). Cyberbullying represents a form of inter-group conflict, such as masculinity vs. femininity (Case 1—education), rich vs. poor (Case 2—entertainment), and home country vs. other country (Case 5—sport). These oppositions feature some degree of power imbalance (Olweus, 1995). People unconsciously classify themselves as either cyberbullies or mediators with respect to cyberbullying; one’s label depends on their identification and understanding of group identity. Case 3 (society) had less cyberbullying content and concerned personal privacy and public safety. Amid the COVID-19 pandemic, a large volume of information about personal trips has come to be stored in the cloud, heightening the risk of privacy breaches. The victim’s experience in this case could have happened to anyone. Posters could therefore naturally assign themselves to the same group as the victim and empathize with him more readily.

Our findings further demonstrated that the sentiment of cyberbullying comments varied by incident. Sentiment entails a comprehensive analysis of the positive, negative, and neutral sentiment in a particular sentence. The proportions of cyberbullying types and the public’s understanding of a controversy each influenced sentiment. When comparing the positive and negative probability in different cyberbullying cases, the positive probability did not differ significantly across cases. This result is interesting but not surprising: implicit bullying was the main type of cyberbullying in each case. Positive and neutral words were widely used to satirize others in all cases, such as “太好笑了” (“That’s funny”) in Cases 3 and 5 or “有钱真好” (“It’s good to be rich”) in Case 2. By contrast, the significant difference in negative probability between Case 1 and others may have emerged because this case included the highest rate of explicit bullying; cyberbullies expressed their anger by swearing extensively and by using negative words and death-related terms. Case 1’s negative probability was accordingly high. The above findings indicate that cyberbullying detection involves more than simply pinpointing negative sentiment (Ptaszynski et al., 2016). Arreerard and Senivongse (2018) found that some text did not express negative opinions although the content was defamatory. In incidents where ridicule was the predominant form of implicit bullying, the proportion of positive emotions was striking. Current approaches to sentiment analysis for cyberbullying detection hence appear insufficient; additional work is needed to ascertain whether a text is extremely emotional.

### Implications for Curbing Cyberbullying and Shaping Digital Citizens

Our results offer several meaningful implications for the detection and governance of cyberbullying. Automatic cyberbullying detection is a task of growing interest and a timely concern given the ubiquity of social networks in everyday life and the potentially dire consequences of cyberbullying (Rosa et al., 2019). This detection tool is correspondingly valuable. However, Rosa et al. (2019) found that cyberbullying is often misrepresented in the literature, leading to inaccurate systems with limited real-world applicability. As discussed, filtering stop words may not increase the accuracy of cyberbullying detection as predicted; more investigation is needed. Existing methods fail to paint a clear picture of cyberbullying; most concentrate on a limited set of textual features to the neglect of other linguistic characteristics (including implicit ones). It is therefore necessary to generate tools capable of more comprehensive textual analysis of cyberbullying. For example, detailed analysis can address comments related to time and relativity, affective and biological processes, pronouns pointing to victims (e.g., “you”), and terms with a negative meaning but positive connotation (i.e., not all bullying content contains explicit insults). It would be similarly useful to detect uncertainty in sentences, as our results frame this lack of certainty as a key component of rumor propagation. Additionally, semantic network analysis reveals words that are most likely to trigger cyberbullying related to a given incident. These terms can then be taken as “seed words” for cyberbullying and introduced into deep learning to improve model accuracy. Second, cyberbullying detection has been expanded to languages other than English. We combined TextMind, with Baidu’s open API of natural language processing, SPSS, and ROSTCM to carry out a preliminary analysis of the linguistic features of Chinese cyberbullying. Our results shed light on the design and development of automatic cyberbullying detection tools for Chinese social networking platforms, such as Sina Weibo, Tencent Weibo, and others.

Regarding the governance of cyberbullying, we recommend that social media platform managers or administrators pay closer attention to comments containing reactions other than explicit aggression: adjunct words (e.g., auxiliary verbs, prepositions, quantifiers, and numbers), frequent assent words, or fillers. In addition, cyberbullies’ preferred forms of cyberbullying and the sentiments expressed varied by case. Platform managers or administrators should therefore tailor their approaches to combating cyberbullying in different areas. For example, in the case involving gender antagonism, platform personnel could screen comments containing stigmatizing epithets that represent men or women. Practitioners can also filter negative comments targeting specific groups. Last, rather than outlining general etiquette for social media users to follow, government officials should establish detailed rules and regulations for separate occasions. They should also aim to clean the cyberspace as thoroughly as possible using advanced technology and monitor sensitive incidents with the potential to garner widespread attention. These interventions can minimize the likelihood of cyberbullying. Further, with these rules, social media platforms should advise their users based on the site’s characteristics. Bilibili, a leading Chinese video streaming website, is one such example. The platform is famous for its bullet comments service that allows real-time comments from viewers to fly across the screen like bullets. Bilibili provides detailed instructions for bullet-comment etiquette; only users who pass the test can send bullet comments.

For individual social media users, the findings of this paper can offer examples of how not to behave elegantly and responsibly on social networks. Users can thus “learn from failure” and reflect on what digital citizenship embodies—what is effective and what is harmful when socializing online. People can hurt others inadvertently, such as by speaking inappropriately. Online interaction leaves clearer footprints than face-to-face communication. These traces are easily tracked and can subject victims to greater pain. Our results confirm this risk: content analysis revealed that people used bullying language despite not intending to speak out against the victims. Ideally, to be an ethical and responsible digital citizen, one should know how to use ICT to build a harmonious and innovative digital society. It is important to bear in mind what is allowed and what is prohibited, when certain behavior is and is not appropriate, and how to respond decently online. Linguistic features can offer users a concrete sense of how to use words properly in various situations on social media. Our research should thus raise users’ awareness of which types of words are preferable to avoid misunderstanding and to discourage cyberbullying. Though anti-cyberbullying is only one of the tasks individuals face while enhancing digital citizenship, it is essential to shaping qualified digital citizens given the prevalence of social networking in daily life. Our research thus makes a meaningful step toward cultivating ethical and responsible digital citizens.

## Conclusion

Cyberbullying has become one of the most challenging problems plaguing social media, as it harms the physical and mental health of individuals as well as online communities. Most relevant research revolves around preventing cyberbullying and has presented data-based approaches to identify such behavior. However, few studies have differentiated between explicit and implicit cyberbullying, leading to unsatisfactory identification results. Moreover, relatively little is known about cyberbullying in non-English language contexts. The present work sought to examine the linguistic features of cyberbullying on Chinese social media. We first reviewed research related to cyberbullying, especially its language and the relationship between cyberbullying and digital citizenship. Then, a systematic linguistic analysis of 23,980 comments from Sina Weibo was conducted, including content analysis, lexical feature analysis, sentiment polarity analysis, and semantic network analysis. We next distinguished the linguistic characteristics of explicit bullying and implicit bullying and examined the prevalence of implicit bullying as well. Results revealed that cyberbullying language follows several patterns. First, comments categorized as implicit bullying (e.g., ridicule and satire) are more prevalent than non-bullying comments. Second, cyberbullying comments generally contain a higher word count, greater number of swear words and adjunct words, heavy use of pronouns (e.g., “you” and “she”), more affective and biological processes, frequent references to money and death, and frequent use of question marks and exclamation points. Third, comments’ sentiment varies by case; the proportion of positive emotions can be underestimated depending on the type of cyberbullying. For instance, cases with more intense conflict in our sample had a higher proportion of explicit cyberbullying language and more negative expressions than others. Finally, the nature of an incident informs word choice: cyberbullying often stems from controversial behavior, leading to attacks on the victim’s appearance, experience, and family as well as potential indiscriminate attacks on a particular group of victims. These findings also underscore the need for cyberbullying research based on pragmatics.

Overall, extracting the linguistic features of cyberbullying contributes to cyberbullying detection and enriches digital citizenship education. The apparent limitations of cyberbullying detection, as indicated by sentiment polarity analysis, reinforce the need for studies identifying the characteristics of implicit bullying and its manifestations. Also, stop words should be used judiciously: words such as prepositions and tense markers, may not be filtered. Regarding digital citizenship education, clear guidelines should be developed for online communication. Qualified digital citizens generally exhibit safe, ethical, and responsible online behavior. The linguistic characteristics of cyberbullying revealed in this study can provide netizens examples of how to communicate properly on social media. Language reflects and influences individuals’ perceptions and behavior (Tausczik and Pennebaker, 2010; Kihlstrom and Park, 2018). A better understanding of online language use can facilitate people’s online interactions, which can in turn change the digital world.

Despite its revelations, this research has limitations that leave room for future work. Emojis were not considered in our analysis of cyberbullying comments; however, the coding process showed that commenters often expressed attack intentions *via* emojis (Miller et al., 2016; Rafi, 2019). We therefore recommend that follow-up research delve further into individuals’ comments, particularly decoding images and emojis (e.g., Hettiarachchi and Ranasinghe, 2019; Kumari and Singh, 2021). Moreover, the linguistic analysis tools adopted in this study were based on LIWC2007 and C-LIWC supplemented with an additional Chinese dictionary. More powerful and updated tools should be applied in subsequent work to accommodate rapidly evolving Chinese Internet vocabulary.

## Data Availability Statement

The original contributions presented in the study are included in the article/supplementary material; further inquiries can be directed to the corresponding authors.

## Author Contributions

JZho: literature search, methodology, data analysis, and writing—original draft preparation and editing. JQ: literature search, data curation, and data analysis. MS: literature search, content analysis, and writing—review and editing. XJ and JZha: content analysis and writing—review and editing. YG: literature search and content analysis. XQ: literature search and content analysis. YX: writing—review and editing. JH: methodology and writing—revision and editing. YZ: supervision, conceptualization, validation, and writing—review and editing. All authors contributed to the article and approved the submitted version.

## Funding

This research was supported by the National Social Science Fund of China under grant no. 21FJKB018, the Postdoctoral Research Foundation of China under grant no. 2021M701273, and the “Challenge Cup” Golden Seed Cultivation Project of South China Normal University under grant no. 21JXKA07.

## Conflict of Interest

The authors declare that the research was conducted in the absence of any commercial or financial relationships that could be construed as a potential conflict of interest.

## Publisher’s Note

All claims expressed in this article are solely those of the authors and do not necessarily represent those of their affiliated organizations, or those of the publisher, the editors and the reviewers. Any product that may be evaluated in this article, or claim that may be made by its manufacturer, is not guaranteed or endorsed by the publisher.
